# New Trends in Freeze-Drying of Pharmaceutical Products

**DOI:** 10.3390/pharmaceutics15071975

**Published:** 2023-07-19

**Authors:** Roberto Pisano, Davide Fissore

**Affiliations:** Dipartimento di Scienza Applicata e Tecnologia, Politecnico di Torino, Corso Duca degli Abruzzi 24, 10129 Torino, Italy; roberto.pisano@polito.it

Freeze-drying, also known as lyophilization, is a process that facilitates the removal of water through sublimation from a frozen product (primary drying). Subsequently, the (low) target value of residual moisture is obtained by promoting desorption of the non-frozen water, bound to the product molecules (secondary drying). Lyophilization is therefore a method of choice for temperature-sensitive products requiring a low water content for shelf stability, preferably without a cold chain commitment, such as biopharmaceutical vaccines, antibiotics, and other products. 

This Special Issue shows the main innovations in the field of freeze-drying of pharmaceutical products, with a specific emphasis (i) on the investigation of formulation features that must be accounted for in case freeze-drying is used as a downstream process, (ii) on the new types of containers being investigated, (iii) on process monitoring in the three steps of the process (freezing, primary drying, and secondary drying), and (iv) on the process innovation in the freezing stage (due to its impact on the drying stages).

The review by Groël et al. [1] addresses the use of differential scanning calorimetry (DSC) and isothermal microcalorimetry (IMC) to investigate, among others, molecular mobility, particularly in the form of α-relaxations, thus assisting in optimizing the formulation recipe. The authors evidenced that an increase in the relaxation time results in higher shelf stability, which may be obtained either through post-processing temper or through collapse or aggressive freeze-drying to remove the excess energy in the product during processing. The optimization of the formulation recipe is also addressed by Vallerinteavide et al. [2], who proposed the use of a nanoscale, thermostable exoshell (tES) to prevent protein aggregation and denaturation in the process.

With respect to the container, alternatives to glass vials were investigated, aiming to obtain, among the other results, better heat transfer to the product in the endothermic stages of the process (primary and secondary drying). Härdter et al. [3] considered containers made of cyclic olefin polymer (COP) as they are characterized by a high break resistance, and they are biocompatible and exhibit homogeneous heat transfer. Unfortunately, they are permeable to gases and, thus, require storage in aluminum pouches in the presence of oxygen and moisture absorbers to perform similarly to glass vial storage. This precaution was not taken by Malik et al. [4], who used only plastic microtubes as an alternative container for small-volume filling. Although they appeared to be a suitable alternative for glycans, they should not be used in the case of labile biological materials.

Product monitoring is a key issue to guarantee product quality in-line, and not by testing at the end of the process, as required by the Guidance for Industry PAT issued by the FDA about two decades ago. Particularly interesting are those techniques that allow the evolution of the product (temperature and residual moisture content) to be monitored in a non-invasive way, without interfering with the process itself. Through-Vial Impedance Spectroscopy (TVIS) was used by Pandya et al. [5] to track the ice sublimation stage, focusing on the change in the shape of the sublimation interface over time and the identification of the end point of primary drying. Always in the field of primary drying, Emteborg et al. [6] used an infrared (IR) thermocamera, placed outside the dryer chamber, to monitor product temperature in a non-invasive way, both at the top surface and vertically, by using custom-made cuvettes, with a Ge window placed close to an IR mirror having a 45° angle. Leys et al. [7] focused on the secondary drying stage, using near-infrared (NIR) spectroscopy, coupled with a PLS-based model, to track the residual moisture of the product vs. time, thus investigating the desorption kinetics and the effect of the operating conditions on the secondary drying stage. Moreover, the researchers pointed out that in the unit used for this study, where a continuous spin freeze-drying process is carried out, using high cooling rates in the freezing stage results in a higher desorption rate, due to the formation of smaller ice crystals.

With respect to the freezing process, which has a substantial impact on the following drying stages as the structure of the dried product relates to the disappearance of the ice crystals, vacuum surface-induced freezing (VISF) was shown by Harguindeguy et al. [8] to promote ice nucleation in the batch at the same time, thus removing the main sources of batch inhomogeneity, both in the case of vials placed over the shelf and in the case of vials being suspended. An IR thermocamera placed inside the drying chamber is used to evaluate the freezing front temperature and its change over time, thus enabling the use of simple models to estimate the distribution of ice crystals. Regis et al. [9] investigated the effect of hydrophilic or hydrophobic inner coatings, pointing out that hydrophobic coatings promoted blow-up and boiling phenomena, while hydrophilic coatings could increase fogging, in all cases resulting in unacceptable aesthetic features in the product. The addition of a surfactant, with a degassing step before VISF, is shown to avoid boiling and bubbling. Nuytten et al. [10] and Lammens et al. [11] investigated spin freezing as a first step in a continuous process for unit doses. A mechanistic model proposed in [10] was used to set and control the vial temperature during freezing, and the impact of the process on the pore size distribution and, finally, on the resistance to mass transfer during primary drying was investigated in [11]. A higher spin-freezing rate results in small lamellar pores, with a low tortuosity structure, while the opposite is obtained at a low spin-freezing rate, with larger, more spherical pores. This is a critical issue as the porous structure has a substantial impact on the drying stages, in particular on the drying kinetics, as shown by Thomik et al. [12], who determined pore structures through micro-computed tomography (μCT), and drying kinetics were obtained through neutron radiographic imaging.

As a concluding remark, we hope that this Special Issue will contribute to highlighting some of the most relevant innovations in the field of freeze-drying of pharmaceutical products, with particular reference to process optimization and monitoring, in order to reduce its cost and guarantee the quality of the final product.
